# Case Report: Methemoglobinemia associated with compound aminopyrine-phenacetin tablets supported by toxicological evidence

**DOI:** 10.3389/fphar.2026.1876072

**Published:** 2026-08-10

**Authors:** Yang Liu, Xue Zhao, Wenqiang Li, Xupeng Shao, Kailiang Fan, Li Kong, Zekun Wei

**Affiliations:** 1 The First Clinical Medical College, Shandong University of Traditional Chinese Medicine, Jinan, Shandong, China; 2 Department of Emergency Center, Affiliated Hospital of Shandong University of Traditional Chinese Medicine, Jinan, Shandong, China

**Keywords:** aminopyrine, drug toxicity, methemoglobinemia, methylene blue, phenacetin, toxicological evidence

## Abstract

**Background:**

Methemoglobinemia (MetHb) is characterized by the oxidation of ferrous iron (Fe2+) in hemoglobin to the ferric state (Fe3+), resulting in impaired oxygen-carrying capacity and subsequent tissue hypoxia. Acquired methemoglobinemia is most commonly induced by drugs or chemical agents; however, it remains relatively rare in clinical practice and is frequently misdiagnosed.

**Case presentation:**

We report a case of drug-induced methemoglobinemia associated with compound aminopyrine-phenacetin tablets (Qutong tablets). An 80-year-old woman with chronic lower limb pain had intermittently used this analgesic prior to admission. She presented with unexplained hypoxemia that was refractory to oxygen therapy. Arterial blood gas analysis revealed a markedly elevated PaO2 (271 mmHg) despite persistently low pulse oximetry saturation, indicating a significant oxygen saturation gap. The MetHb level was 22.4%. Toxicological testing detected plasma aminopyrine and phenacetin concentrations above the laboratory reference limits, documenting relevant drug exposure and supporting the clinical attribution in the context of the medication history, saturation gap, elevated MetHb level, and treatment response. Following discontinuation of the suspected agent and administration of methylene blue combined with vitamin C, MetHb levels rapidly normalized and hypoxemia significantly improved.

**Conclusion:**

This case highlights that chronic, irregular exposure to oxidant drugs may be associated with clinically significant methemoglobinemia even in the absence of a clear acute overdose. Clinicians should maintain a high index of suspicion in patients with hypoxemia unresponsive to oxygen therapy and evidence of an oxygen saturation gap. Careful medication history, co-oximetry, and toxicological testing may help document exposure and support diagnostic attribution in clinically compatible cases. Keywords: methemoglobinemia; phenacetin; aminopyrine; methylene blue; drug toxicity; toxicological evidence.

## Introduction

Methemoglobinemia is a pathological condition in which the iron within hemoglobin is oxidized from the ferrous (Fe2+) to the ferric (Fe3+) state, thereby impairing its ability to bind and transport oxygen (Ash-Bernal et al., 2004; Umbreit, 2007). Under normal physiological conditions, methemoglobin levels are maintained below 1% through the action of the NADH-dependent cytochrome b5 reductase system (Umbreit, 2007; Percy and Lappin, 2008). However, excessive exposure to exogenous oxidizing agents may overwhelm this endogenous reduction capacity, resulting in the accumulation of methemoglobin and subsequent tissue hypoxia (Ash-Bernal et al., 2004; Bradberry et al., 2001).

Acquired methemoglobinemia is most commonly associated with exposure to oxidant drugs or chemicals, including local anesthetics, nitrates, sulfonamides, and aromatic amine derivatives (Bradberry et al., 2001; Wright et al., 1999). Among these, aromatic amines are of particular clinical relevance, as their metabolic products possess strong oxidative properties capable of converting hemoglobin iron into its ferric form Bradberry et al. (2001), Coleman and Coleman (1996).

Compound aminopyrine-phenacetin tablets (Qutong tablets) are a combination analgesic formulation containing aminopyrine, phenacetin, caffeine, and phenobarbital. Phenacetin, in particular, has been documented to induce methemoglobin formation through oxidative metabolism, potentially leading to cyanosis and hypoxemia (Coleman and Coleman, 1996). Although the clinical use of this compound has declined in many regions, it remains accessible in certain settings.

In this report, we describe a case of methemoglobinemia induced by Qutong tablets and discuss the possible pharmacological mechanisms and clinical implications, with the aim of improving recognition of this uncommon but potentially life-threatening condition.

## Case description

An 80-year-old woman with a history of peripheral arterial occlusive disease, coronary artery disease, hypertension, chronic kidney insufficiency, hypothyroidism, and paroxysmal atrial fibrillation was admitted due to progressively worsening bilateral lower limb edema and pain over 2 months, accompanied by altered consciousness 1 day prior to admission. The patient had a longstanding history of lower limb pain associated with peripheral arterial disease and had intermittently self-administered over-the-counter analgesics for symptom relief.

Upon admission, the patient exhibited significant hypoxemia, with a pulse oximetry saturation of approximately 85%. Respiratory failure was initially suspected, and supportive oxygen therapy was administered; however, oxygenation did not improve significantly. Physical examination of the lungs did not reveal obvious abnormalities, suggesting that conventional respiratory causes could not fully account for the patient’s hypoxic state.

## Diagnostic assessment

Arterial blood gas analysis performed on 10 March 2026, revealed a pH of 7.41, PaCO2 of 24.4 mmHg, and a markedly elevated PaO2 of 271 mmHg, with a P/F ratio of 542 mmHg. Despite the elevated PaO2, pulse oximetry saturation remained persistently low, indicating a pronounced oxygen saturation gap. The methemoglobin level was measured at 22.4%, and the arterial blood exhibited a characteristic chocolate-brown discoloration. These findings strongly suggested impaired oxygen delivery due to methemoglobinemia.

Further inquiry into the patient’s medication history revealed that she had been intermittently taking compound aminopyrine-phenacetin tablets for approximately 2 months prior to admission. Each tablet contained aminopyrine 150 mg, phenacetin 150 mg, caffeine 50 mg, and phenobarbital 15 mg. Although the patient typically consumed one tablet per day, she reported occasional dose escalation during episodes of severe pain. Because the patient had used the tablets irregularly, the exact timing of the last ingestion and the cumulative dose could not be reliably determined.

**FIGURE 1:**
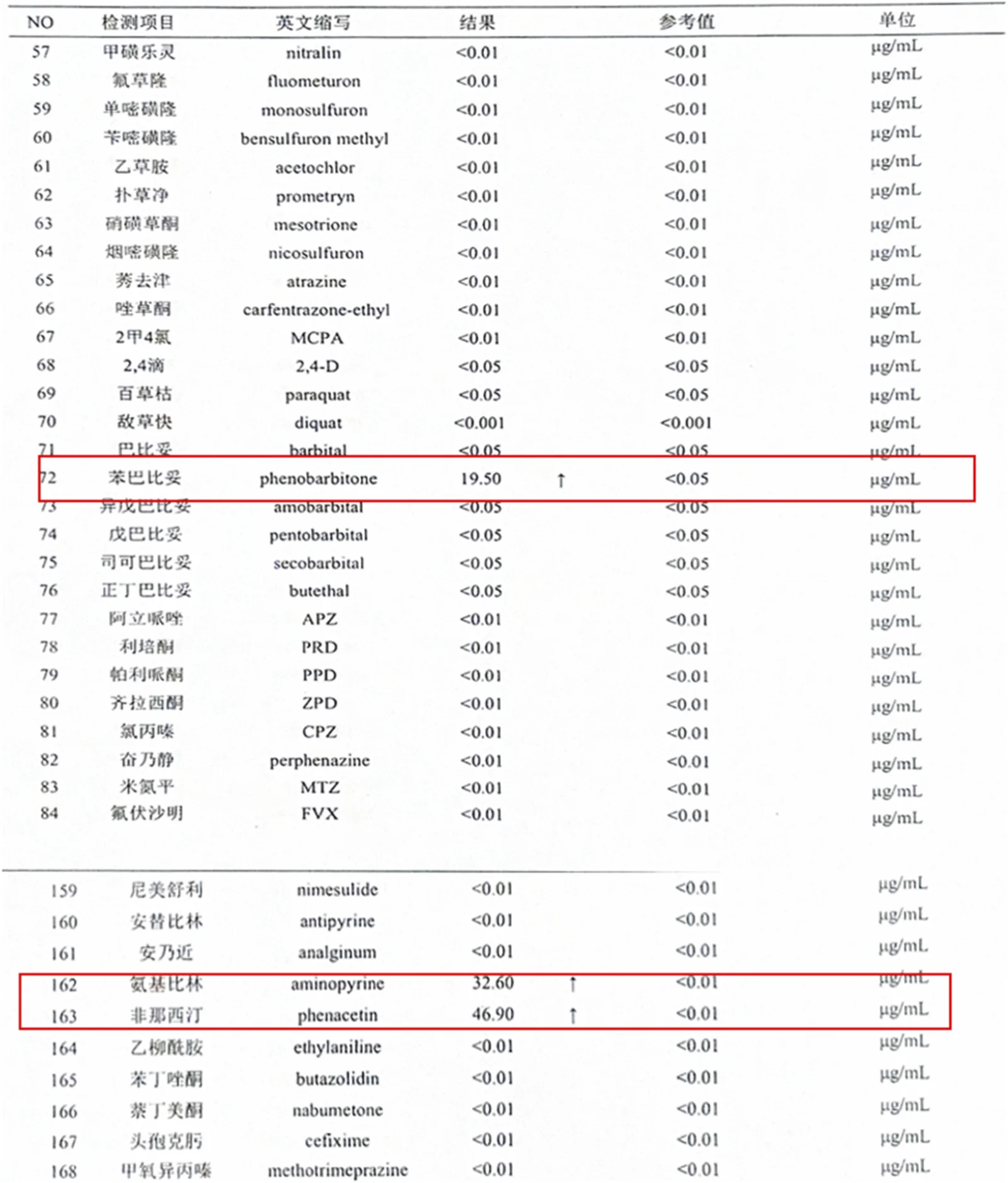
Toxicological screening results documenting plasma exposure to phenobarbitone, aminopyrine, and phenacetin. The patient’s plasma sample was collected on 10 March 2026, received by the laboratory on 12 March 2026, and reported on 13 March 2026. Toxicological testing was performed using liquid chromatography–tandem mass spectrometry as part of a 252-compound unknown-poison screening panel. The analysis detected phenobarbital/phenobarbitone (19.50 μg/mL), aminopyrine (32.60 μg/mL), and phenacetin (46.90 μg/mL), all above the laboratory reporting/reference limits. Aminopyrine and phenacetin are known oxidant-related agents capable of contributing to methemoglobin formation. These findings document exposure to components of compound aminopyrine-phenacetin tablets and should be interpreted together with the medication history, MetHb level, oxygen saturation gap, and treatment response.

Given the known oxidative properties of aminopyrine and phenacetin, along with the patient’s history of prolonged and irregular use, drug-induced methemoglobinemia was strongly suspected. A plasma sample collected on 10 March 2026, was submitted for toxicological testing using liquid chromatography–tandem mass spectrometry as part of a 252-compound unknown-poison screening panel. The analysis detected aminopyrine (32.60 μg/mL), phenacetin (46.90 μg/mL), and phenobarbitone (19.50 μg/mL), all above the laboratory reference limits (Figure 1). These findings documented exposure to components of Qutong tablets but were interpreted together with the clinical presentation rather than as stand-alone proof of toxic accumulation or causality. Based on the saturation gap, elevated MetHb level, characteristic blood discoloration, medication history, clinical response, and toxicological evidence of exposure, a probable diagnosis of drug-induced methemoglobinemia associated with Qutong tablet use was made. The Naranjo Adverse Drug Reaction Probability Scale yielded an estimated score of 7, consistent with a probable adverse drug reaction. The patient’s clinical course is summarized in Table 1.

**TABLE 1:** Clinical timeline of the episode of care.

Time	Event
2 months before admission	Intermittent use of analgesic (Qutong tablets)
1 day before admission	Onset of altered consciousness
Day 0	Admission with hypoxemia
Day 0	ABG: MetHb 22.4%
Day 0	First methylene blue administration
Day 1	Second methylene blue administration
Day 2	MetHb decreased to 0.6%

## Therapeutic intervention

The suspected medication was immediately discontinued, and treatment with methylene blue in combination with vitamin C was initiated. The patient received 120 mg of methylene blue and 0.5 g of vitamin C on March 10, followed by a second identical dose on March 11. The dose of methylene blue was consistent with commonly recommended dosing for symptomatic methemoglobinemia. Glucose-6-phosphate dehydrogenase testing was not available before treatment. Given the presence of symptomatic methemoglobinemia, persistent hypoxemia despite oxygen therapy, and an elevated MetHb level, immediate treatment was considered necessary after weighing the risk of ongoing tissue hypoxia against the potential risk of hemolysis. The patient was monitored clinically during and after therapy, and no overt hemolytic event was observed. Nevertheless, the absence of pre-treatment G6PD testing should be acknowledged as a limitation, and post-stabilization testing may be considered when feasible.

## Follow-up and outcomes

**FIGURE 2:**
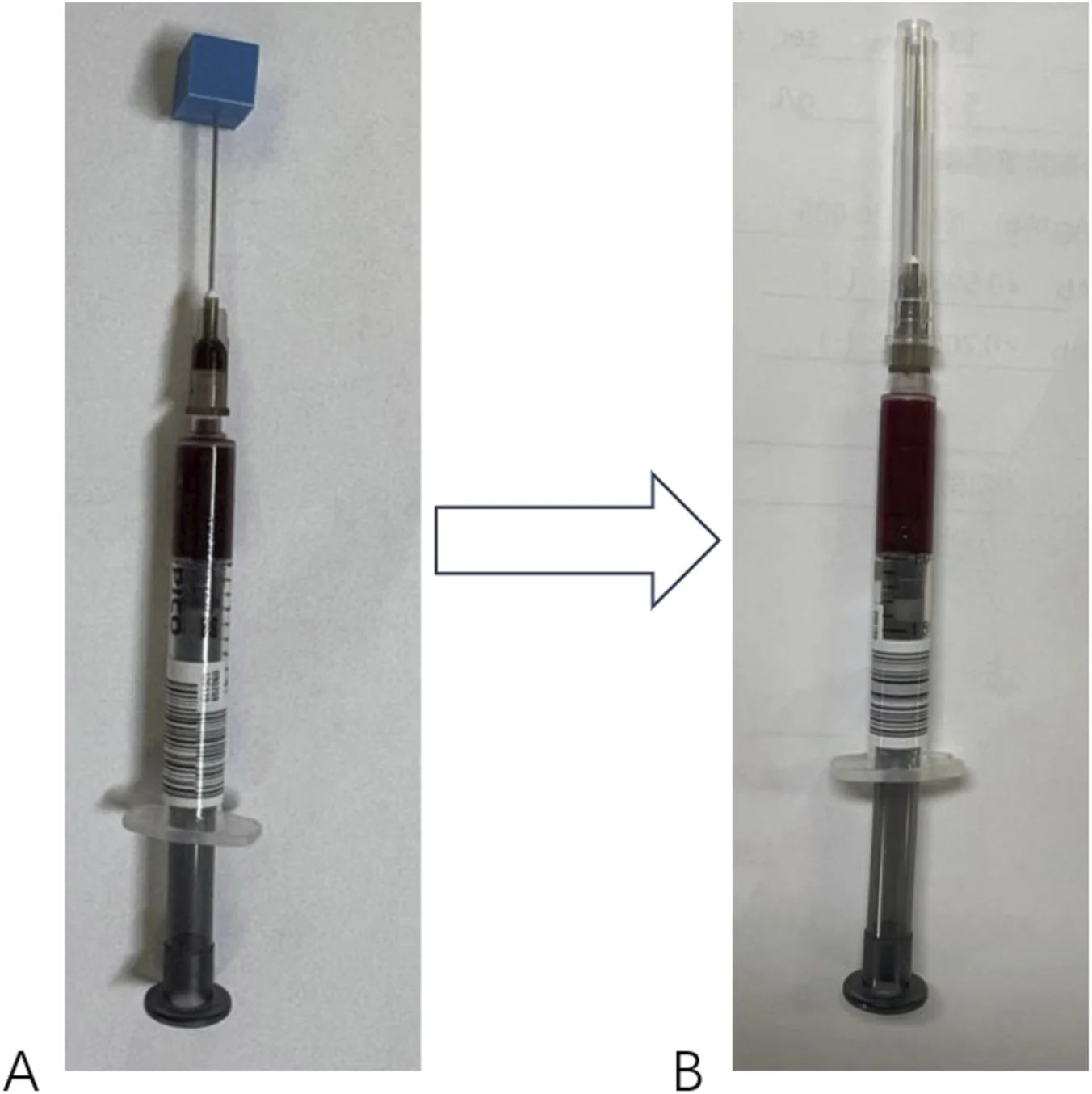
Macroscopic changes in arterial blood color before and after treatment. **(A)** Arterial blood sample obtained before treatment showing a characteristic chocolate-brown discoloration, consistent with elevated methemoglobin levels. **(B)** Arterial blood sample obtained after methylene blue administration demonstrating restoration of normal bright red color, indicating improvement in hemoglobin oxygen-carrying capacity.

**FIGURE 3:**
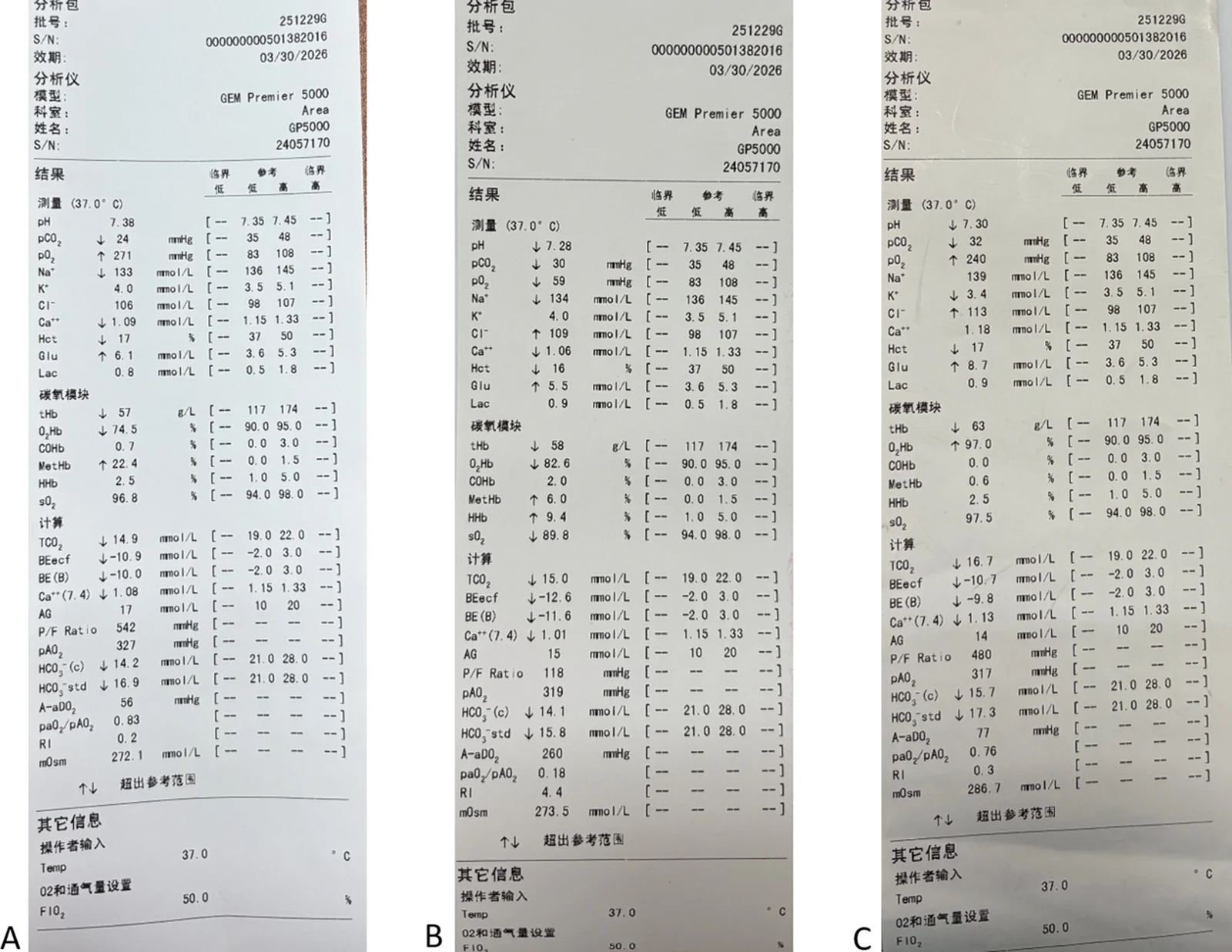
Dynamic changes in arterial blood gas parameters and methemoglobin levels following treatment. **(A)** Initial arterial blood gas analysis performed on March 10, 2026 before treatment, demonstrating severe methemoglobinemia with a marked oxygen saturation gap. **(B)** Repeat arterial blood gas analysis on March 11, 2026 after the first administration of methylene blue, showing a marked decrease in methemoglobin level. **(C)** Follow-up arterial blood gas analysis on March 12, 2026 demonstrating normalization of methemoglobin level and improvement in oxygenation.

Following treatment, the patient developed dark green urine, consistent with methylene blue metabolism, and the color of arterial blood gradually changed from chocolate-brown to bright red (Figure 2). Repeat measurements demonstrated a rapid decline in MetHb levels to 6.0% on March 11 and further to 0.6% on March 12 (Figure 3), accompanied by marked improvement in hypoxemia and overall clinical status.

## Discussion

Drug-induced methemoglobinemia arises when the oxidative burden imposed by exogenous agents exceeds the capacity of endogenous enzymatic reduction systems (Ash-Bernal et al., 2004; Bradberry et al., 2001). Under physiological conditions, the NADH-dependent cytochrome b5 reductase pathway serves as the primary mechanism for maintaining methemoglobin at low levels (Umbreit, 2007; Percy and Lappin, 2008). However, exposure to strong oxidants may disrupt this balance, leading to the accumulation of methemoglobin and impaired oxygen delivery (Ash-Bernal et al., 2004; Bradberry et al., 2001).

Aromatic amine derivatives represent a major class of agents implicated in acquired methemoglobinemia (Bradberry et al., 2001; Coleman and Coleman, 1996). Both phenacetin and aminopyrine, the principal oxidant components of Qutong tablets, undergo hepatic metabolism to generate reactive oxidative intermediates capable of converting hemoglobin iron from the ferrous to the ferric state. In the present case, toxicological analysis detected these compounds in plasma at concentrations above the laboratory reference limits, providing objective evidence of exposure to oxidant-related components in a clinically compatible setting.

In addition to these oxidant components, the formulation also contains phenobarbital, a known inducer of several cytochrome P450 enzymes (Zanger and Schwab, 2013). Therefore, a phenobarbital-mediated increase in oxidative metabolite formation from aminopyrine or phenacetin is pharmacologically plausible (Zanger and Schwab, 2013; Guengerich, 2008). However, no metabolic assays, serial drug concentrations, or enzyme activity measurements were performed in this patient; this mechanism should therefore be regarded as a hypothesis rather than a demonstrated causal pathway. Such a potential interaction may be clinically relevant in vulnerable populations, including elderly patients or those with impaired renal function, but further pharmacokinetic evidence is needed.

From a clinical perspective, methemoglobinemia should be considered in patients presenting with unexplained hypoxemia that does not respond adequately to oxygen therapy (Wright et al., 1999; Skold et al., 2011). A key diagnostic feature is the presence of an oxygen saturation gap, characterized by a discrepancy between low pulse oximetry saturation and high arterial oxygen tension. The characteristic chocolate-brown appearance of arterial blood may provide an additional visual clue (Ash-Bernal et al., 2004; Skold et al., 2011). Several alternative causes of refractory low pulse oximetry saturation should also be considered. Primary pulmonary causes of hypoxemia were less likely in this patient because arterial oxygen tension was markedly elevated despite persistently low pulse oximetry saturation, indicating a saturation gap. Carbon monoxide poisoning and other dyshemoglobinemias may also produce discordance between oxygen saturation measurements and clinical oxygenation status; therefore, exposure history and co-oximetry findings should be reviewed when available. Sulfhemoglobinemia was another potential consideration, but the elevated measured MetHb level, characteristic chocolate-brown arterial blood, and rapid biochemical and clinical response after methylene blue made methemoglobinemia the most likely explanation. Pulse oximeter artifact and hypoperfusion-related measurement error were also considered, but the persistent saturation gap and reproducible clinical findings supported a true dyshemoglobinemia rather than isolated measurement error.

The mainstay of treatment for drug-induced methemoglobinemia involves prompt discontinuation of the offending agent and administration of methylene blue in symptomatic patients or those with significantly elevated MetHb levels (Wright et al., 1999; Skold et al., 2011). Methylene blue acts as an electron carrier in the NADPH-dependent reduction pathway, facilitating the conversion of ferric iron back to its functional ferrous state (Umbreit, 2007; Skold et al., 2011). Adjunctive use of vitamin C may further enhance the reduction of methemoglobin. Recent case reports have also demonstrated that combined therapy with methylene blue and vitamin C can effectively reduce MetHb levels and improve clinical outcomes (Pjetraj et al., 2025). In the present case, the rapid decline in MetHb levels and clinical improvement following treatment further support the effectiveness of this therapeutic approach.

Several limitations should be acknowledged. First, although toxicological testing documented plasma exposure to aminopyrine, phenacetin, and phenobarbitone at admission, serial measurements were not performed after clinical improvement, limiting the ability to characterize drug clearance kinetics. Second, because the patient had used the medication irregularly, the exact timing of the last ingestion and the cumulative dose could not be precisely quantified. Third, the laboratory report provided reference limits rather than disease-specific toxic thresholds or pharmacokinetic reference ranges; therefore, the toxicological results should be interpreted as supportive evidence of exposure rather than definitive proof of toxic accumulation. Fourth, G6PD testing was not available before methylene blue administration, although immediate treatment was considered necessary because of symptomatic methemoglobinemia and persistent hypoxemia. Finally, not all alternative dyshemoglobinemias, such as sulfhemoglobinemia, could be fully excluded by dedicated testing. Future studies incorporating dynamic drug monitoring and more comprehensive dyshemoglobinemia assessment may provide additional insights into the pharmacokinetic and toxicological mechanisms involved.

Compared with many previously reported cases associated with acute or high-dose exposure (Pjetraj et al., 2025; Ali et al., 2025; Malik et al., 2023), the present case emphasizes chronic irregular analgesic use in an elderly patient with multiple comorbidities. It reminds clinicians that clinically significant methemoglobinemia may occur after repeated intermittent exposure in susceptible patients, even without a clearly defined acute overdose. In addition, quantitative toxicological testing documented exposure to aminopyrine, phenacetin, and phenobarbitone, which supports the clinical attribution when interpreted together with the medication history, saturation gap, elevated MetHb level, and treatment response.

A recent Chinese case report described methemoglobinemia associated with poisoning from compound aminopyrine-phenacetin tablets, in which the MetHb level was 24.3% before treatment and decreased after methylene blue and vitamin C therapy (Sun et al., 2025). That report emphasized acute poisoning and dynamic changes in oxygenation parameters. In contrast, the present case was characterized by chronic irregular analgesic use in an elderly patient with multiple comorbidities, rather than a clearly defined acute overdose. The additional toxicological testing in our case documented plasma exposure to aminopyrine, phenacetin, and phenobarbitone, which supports but does not independently prove the clinical attribution.

### Pharmacological implications

This case underscores the pharmacological risks associated with aromatic amine-containing analgesics. Phenacetin and aminopyrine are capable of generating oxidative metabolites that convert hemoglobin iron into the ferric state, thereby impairing oxygen transport. Moreover, the presence of phenobarbital as a cytochrome P450 inducer raises the possibility of altered oxidative metabolism within this combination formulation. However, this potential interaction was not directly demonstrated in the present patient and should be interpreted as a pharmacologically plausible hypothesis. These observations suggest that combination formulations containing oxidant drugs and enzyme-inducing agents may warrant particular caution in susceptible populations.

## Key learning points

1. Methemoglobinemia should be suspected in patients with hypoxemia unresponsive to oxygen therapy, particularly when an oxygen saturation gap is present. A discrepancy between low pulse oximetry saturation and elevated arterial oxygen tension represents a critical diagnostic clue that should prompt further evaluation.
2. Chronic, irregular, and intermittent exposure to oxidant drugs may be associated with clinically significant methemoglobinemia even in the absence of a clearly defined acute overdose. This case reminds clinicians to consider repeated non-standard analgesic use as a possible risk factor, especially in elderly patients with comorbidities.
3. Combination formulations containing aromatic amines may increase the risk of methemoglobinemia through oxidative metabolites. The presence of cytochrome P450 inducers, such as phenobarbital, may theoretically influence oxidative metabolite formation, but this potential interaction requires further pharmacokinetic evidence.
4. Toxicological analysis can provide objective evidence of exposure to suspected agents and support diagnostic attribution when interpreted alongside medication history, co-oximetry findings, MetHb levels, and treatment response. Quantitative detection of suspected agents in plasma may be particularly helpful in complex clinical scenarios.
5. Prompt discontinuation of the suspected agent and timely administration of methylene blue remain the cornerstone of effective management. Early recognition and intervention are essential for rapid reversal of methemoglobinemia and improved patient outcomes.

## Conclusion

Compound aminopyrine-phenacetin tablets may be associated with acquired methemoglobinemia, especially in elderly patients or those with irregular medication use. Clinicians should maintain a high index of suspicion in patients presenting with unexplained hypoxemia, poor response to oxygen therapy, and evidence of an oxygen saturation gap. Early recognition, discontinuation of the suspected agent, and prompt treatment with methylene blue when indicated are essential for rapid reversal of methemoglobinemia and improved clinical outcomes.

## Data Availability

The original contributions presented in the study are included in the article/Supplementary Material, further inquiries can be directed to the corresponding authors.
