# Supplementary material for: Case Report: Methemoglobinemia associated with compound aminopyrine-phenacetin tablets supported by toxicological evidence
Source: Front Pharmacol. 2026 Aug 10;17:1876072. doi: 10.3389/fphar.2026.1876072 (PMC13500310; doi:10.3389/fphar.2026.1876072)
Supplement: Supplementary file 1 [file DataSheet1.pdf]

CARE case report guidelines

CARE Checklist of information to include when writing a case report

(cc) BY-NC-ND

Topic	Item	Checklist item description	Reported on Line
Title	1	The diagnosis or intervention of primary focus followed by the words "case report"	Report (TItle page)
Key Words	2	2 to 5 key words that identify diagnoses or interventions in this case report, including "case report"	Reported (Keywords section)
Abstract (no references)	3a	Introduction: What is unique about this case and what does it add to the scientific literature?	Reported (Abstract)
	3b	Main symptoms and/or important clinical findings	Reported (Abstract)
	3c	The main diagnoses, therapeutic interventions, and outcomes	Reported (Abstract)
	3d	Conclusion—What is the main "take-away" lesson(s) from this case?	Reported (Abstract conclusion)
Introduction	4	One or two paragraphs summarizing why this case is unique (**may include references**)	Reported (Introduction)
Patient Information	5a	De-identified patient specific information.	Reported (Case Description)
	5b	Primary concerns and symptoms of the patient.	Reported (Case Description)
	5c	Medical, family, and psycho-social history including relevant genetic information	Reported (Case Description)
	5d	Relevant past interventions with outcomes	Reported (Case Description)
Clinical Findings	6	Describe significant physical examination (PE) and important clinical findings.	Reported (Case Description)
Timeline	7	Historical and current information from this episode of care organized as a timeline	Reported (Table 1)
Diagnostic Assessment	8a	Diagnostic testing (such as PE, laboratory testing, imaging, surveys).	Reported (Diagnostic Assessment)
	8b	Diagnostic challenges (such as access to testing, financial, or cultural)	Not applicable
	8c	Diagnosis (including other diagnoses considered)	Reported (Diagnostic Assessment)
	8d	Prognosis (such as staging in oncology) where applicable	Not applicable
Therapeutic Intervention	9a	Types of therapeutic intervention (such as pharmacologic, surgical, preventive, self-care)	Reported (Therapeutic Intervention)
	9b	Administration of therapeutic intervention (such as dosage, strength, duration)	Reported (Therapeutic Intervention)
	9c	Changes in therapeutic intervention (with rationale)	Reported (Therapeutic Intervention)
Follow-up and Outcomes	10a	Clinician and patient-assessed outcomes (if available)	Reported (Follow-up and Outcomes)
	10b	Important follow-up diagnostic and other test results	Reported (Follow-up and Outcomes)
	10c	Intervention adherence and tolerability (How was this assessed?)	Reported (Follow-up and Outcomes)
	10d	Adverse and unanticipated events	Reported (Follow-up and Outcomes)
Discussion	11a	A scientific discussion of the strengths AND limitations associated with this case report	Reported (Discussion)
	11b	Discussion of the relevant medical literature **with references**.	Reported (Discussion)
	11c	The scientific rationale for any conclusions (including assessment of possible causes)	Reported (Discussion)
	11d	The primary "take-away" lessons of this case report (without references) in a one paragraph conclusion	Reported (Conclusion and Key Learning Points)
Patient Perspective	12	The patient should share their perspective in one to two paragraphs on the treatment(s) they received	Not available
Informed Consent	13	Did the patient give informed consent? Please provide if requested	Yes ☑ No ☐
